# The Wuhan-Zhuhai (WHZH) cohort study of environmental air particulate matter and the pathogenesis of cardiopulmonary diseases: study design, methods and baseline characteristics of the cohort

**DOI:** 10.1186/1471-2458-14-994

**Published:** 2014-09-24

**Authors:** Yuanchao Song, Jian Hou, Xiji Huang, Xiaomin Zhang, Aijun Tan, Yi Rong, Huizhen Sun, Yun Zhou, Xiuqing Cui, Yuqing Yang, Yanjun Guo, Zhihong Zhang, Xin Luo, Bing Zhang, Fan Hou, Xiaosheng He, Jungang Xie, Tangchun Wu, Weihong Chen, Jing Yuan

**Affiliations:** Department of Occupational and Environmental Health, School of Public Health, Tongji Medical College, Huazhong University of Science and Technology, Wuhan, 430030 China; Key Laboratory of Environment and Health in Ministry of Education & Ministry of Environmental Protection, and State Key Laboratory of Environmental Health (Incubating), School of Public Health, Tongji Medical College, Huazhong University of Science and Technology, Wuhan, 430030 China; Zhuhai Center for Disease Control & Prevention, Zhuhai, China; Department of Respiratory and Critical Care Medicine, Tongji Hospital, Tongji Medical College, Huazhong University of Science and Technology, Wuhan, China

**Keywords:** Cohort study, Air pollutants, Particulate matter, Pulmonary function, Respiratory diseases, Cardiovascular diseases

## Abstract

**Background:**

Particulate air pollution has been recognized to be associated with a wide range of adverse health effects, including increased mortality, morbidity, exacerbation of respiratory conditions. However, earlier physiological or pathological changes or long-term bodies’ reaction to air pollutants have not been studied in depth in China. The Wuhan-Zhuhai (WHZH) cohort study is designed to investigate the association between air pollutants exposure and physiological or pathological reactions on respiratory and cardiovascular system.

**Methods/Design:**

The cohort is a community-based prospective study that includes 4812 individuals aged 18–80 years. The collections of data were conducted from April to May 2011 in Wuhan city and in May 2012 in Zhuhai city. At baseline, data on demographic and socioeconomic information, occupational history, family disease history, lifestyle, cooking mode, daily travel mode, physical activity and living condition have been collected by questionnaires. Participants underwent an extensive physical examination, including anthropometry, spirometry, electrocardiography, and measurements of blood pressure, heart rate, exhaled nitric oxide and carbon monoxide. Potential conditions in the lung, heart, liver, spleen, and skin were synchronously performed. In addition, samples of morning urine, fasting blood serum and plasma were collected during physical health examination. DNA were extracted and were stored at -80°C. Environment concentrations of particulate matter and chemicals were determined for 15 days in each of four seasons. Participants are followed for physiological or pathological changes or incidence of cardiopulmonary diseases every 3 years.

**Discussion:**

The results obtained in WHZH cohort study may increase a better understanding of the relationship between particulate air pollution and its components and possible health damages. And the potential mechanisms underlying the development of cardiopulmonary diseases has implications for the development of prevention and treatment strategies.

## Background

Air pollution, especially particulate matter (PM) and its components have been recognized to be associated with a wide range of adverse health effects, including increased mortality, elevated rates of hospital admissions and emergency department visits and exacerbation of chronic respiratory conditions by recent reports [1, 2]. In North America and Europe, several cohort studies have been conducted to evaluate the adverse health effects of ambient PM pollution [3–8]. Since the characteristics and components of ambient air pollution, as well as socio-demographic status of local residents in developing countries that may be different from developed countries, the exposure-response coefficients of air pollutants and health damage observed in the developed studies could not simply be applied to the developing countries [9]. Relatively high levels of environmental PM and associated pathophysiological changes in developing countries need further evaluation.

China has experienced rapid industrialization, urbanization and urban transportation development in the past 20 years. During the developing process, industrial emissions, urban construction and increased vehicle exhausts led to poor air quality in many cities in China. The air qualities in some cities were thought to be worst in the world [10, 11]. With the increasing levels of air PM, their health impacts and consequent disease burden have become a growing concern [12]. Current time-series and case-crossover studies conducted in China’s larger cities suggested that short-term exposure to air pollutants was associated with elevated mortality and morbidity from respiratory and cardiovascular diseases [13, 14]. However, earlier physiological or pathological changes or long-term bodies’ reaction to particulate air pollution have not been studied in depth. In addition, the proportion of never-smokers who develop chronic obstructive pulmonary disease (COPD) and lung cancer in China was much higher than those in the most other countries [15, 16]. New results in genome-wide association studies have identified a large number of variants for respiratory diseases including lung function decline and cardiovascular diseases [17–23]. For example, the rs8034191 C allele and PM from cigarette smoking were contributed to the development of COPD [24]. Unfortunately, the adverse effects of PM as well as genetic contribution to health of the Chinese population have yet not been clearly determined. The cities in China, as relative severe PM pollution areas, provide a unique opportunity to examine the direct reactions and adaptations of the body in response to PM and evaluate the potential role of gene-PM interactions in the process of health damage.

The Wuhan-Zhuhai (WHZH) cohort study, an ongoing community-based cohort study, is designed to investigate the association between long-term particulate air pollution exposure and its components and physiological or pathological reactions or health damages on respiratory and cardiovascular system. As the study endpoints, lung function declined, changes of heart rate variability (HRV), hypertension, dyslipidemia, cardiovascular diseases, COPD, and asthma will be studied. In addition, other markers of inflammation and oxidative stress, such as exhaled nitric oxide (NO) [25] and exhaled carbon monoxide (CO) [26, 27] will also be evaluated. And, the gene-PM interactions in human health will be evaluated though personal PM exposure assessment and genetic polymorphisms determinants.

## Methods/Design

### Study area and population

**Figure 1:**
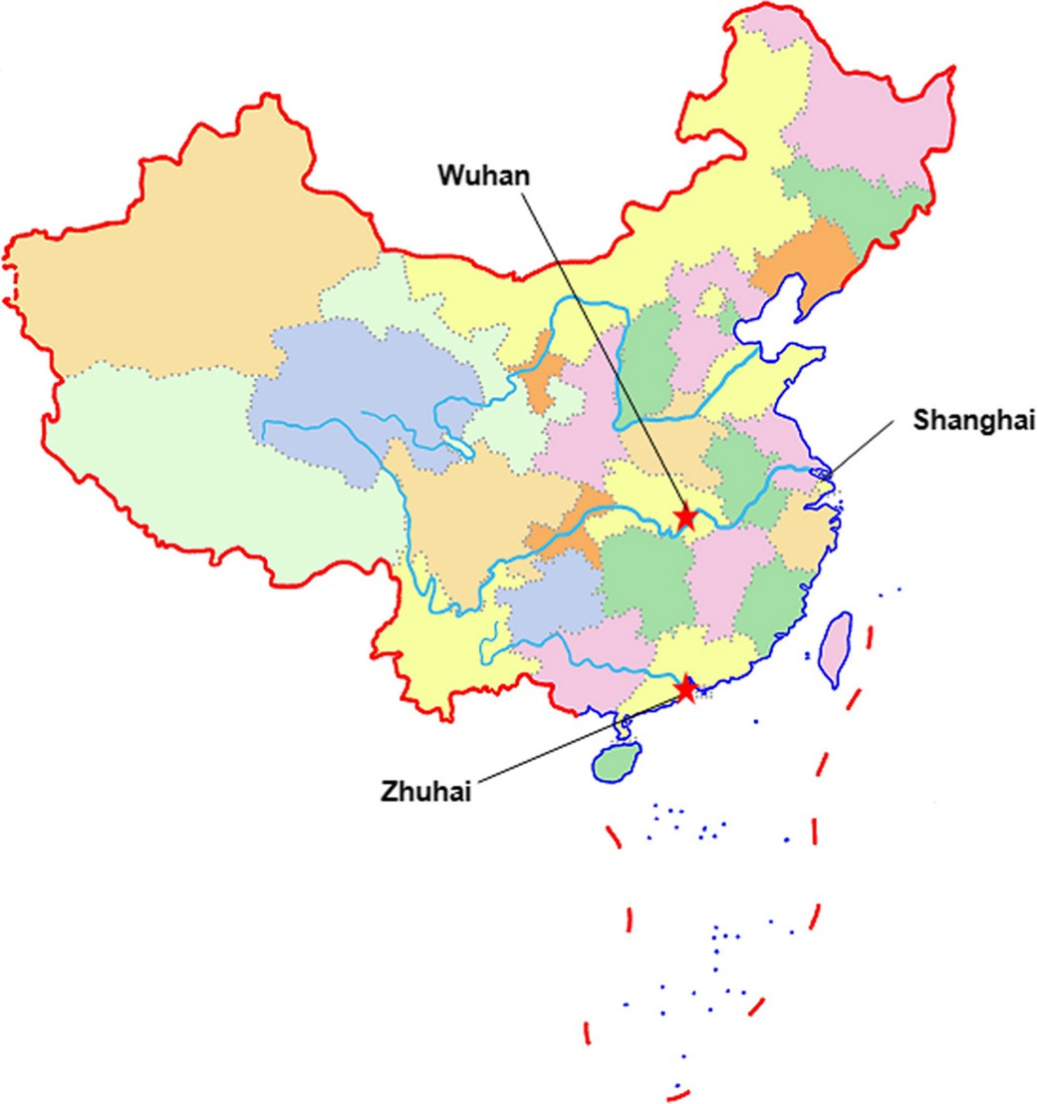
**Geographical locations of the selected cities in the WHZH cohort study.**

**Figure 2:**
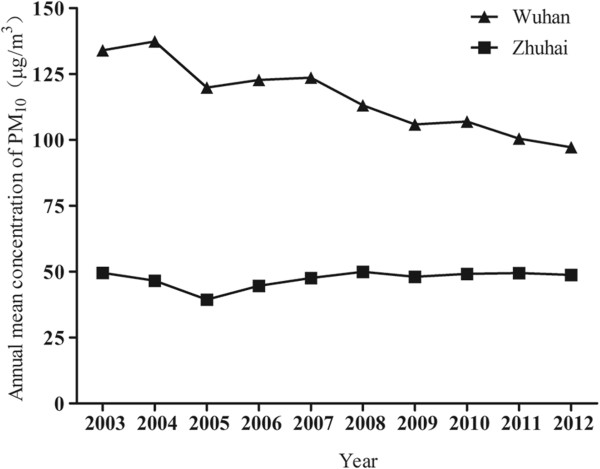
**Annual mean concentration of PM**_**10**_ **in the two cities from 2003 to 2012.**

The WHZH study was conducted in Wuhan and Zhuhai city (Figure 1) in China. The two cities were carefully selected based on significant difference in annual mean concentrations of PM_10_ (particles with aerodynamic diameters less than 10 μm) in the past 10 years (Figure 2). Wuhan, the capital of Hubei province and the largest city in central China, covers an area of 8476 square kilometers, and with a population of eight million. Annual mean PM_10_ concentrations in Wuhan ranged from 97 μg/m^3^ to 137 μg/m^3^ with mean concentration of 116 μg/m^3^ during 2003 to 2012. Annual mean PM_10_ concentrations gradually decreased in the past several years, but the lowest annual PM_10_ concentration was still higher than 70 μg/m^3^, the interim target -1of air quality PM_10_ recommended by the World Health Organization (WHO) [28]. Zhuhai is a gardenlike coastal city of Guangdong province in Southern China, and covers an area of 1701 square kilometers, and with a population of one million. Annual mean PM_10_ concentrations in Zhuhai city were from 39 μg/m^3^ to 50 μg/m^3^ with mean concentration of 47 μg/m^3^ during 2003–2012, and little lower than interim target -2 (50 μg/m^3^) recommended by WHO [28]. A stratified, cluster sampling approach was used to select study communities. As a result, two communities (one in urban district, one in suburb) were selected in each city, respectively.

All residents in the study communities aged between 18 and 80 years, living in the sampling buildings for more than 5 years are eligible to participate in the WHZH study. Nevertheless, these residents are excluded if they had severe illnesses, and were unable to attend clinic visits.

The study protocol was approved by the Medical Ethics Committee of the School of Public Health, Tongji Medical College, HUST. All eligible residents were given a detailed description on the study, in addition to an oral explanation at the study site. All participants have given written informed consent for participation, for storage and use of blood and urine samples, and for obtaining medical records information during follow-up.

The recruitment started in April and completed in May 2011, Wuhan city, and the same work was done in May 2012, Zhuhai city. In total, 4812 participants have been included. In addition, a subgroup included 240 participants between 40 to 60 years old (60 participants in each community) were convened from the cohort participants for 24-hour HRV and 24-hour personal PM exposure determination.

### Study design

The WHZH study is a community-based, prospective cohort study of individuals aged 18 to 80 years. Each study community has a community office that is responsible for the daily routine service. In this study, the members in community office facilitate the communication with all participants of the cohort. Participants were invited to come to the health center in the community for questionnaires and physical examination after an overnight fast. After obtaining written informed consent, a semi-structured questionnaire was used to collect baseline data by trained interviewers. The general health examination was synchronously performed by trained physicians, nurses and technicians. Fasting blood and morning urine sample were collected for measurements of biochemical traits (Table 1). In addition, clinical examinations were conducted for potential conditions in the lung, heart, liver, spleen, and skin.

**Table 1:** **Summary of data items in the WHZH cohort**

Category	Measurements
Demographics and socioeconomics	Birthday, residential address, race, religion, housing conditions, household income, education, work status, occupation, marital status
Life style	Diet, smoking, alcohol consumption, tea, physical activity, sleep, afternoon nap, pets, plants
Family history of diseases	Family history of hypertension, hyperlipidemia, coronary heart disease, diabetes, stroke, cancers, emphysema, chronic bronchitis, asthma, pulmonary tuberculosis, chronic hepatitis, nephritis and arthritis
Past medical history	Diagnosed medical conditions, use of health services, use of medicines for the most recent two weeks
Environmental exposure	Occupational history, environmental tobacco smoke, exposure to traffic, cooking and heating mode, the use of exhaust fan
Anthropometrics	Height, weight, waist circumference, hip circumference
Clinical test	Blood pressure, heart rate, lung function, heart rate variability, exhaled nitric oxide, exhaled carbon monoxide, cardiopulmonary auscultation, smell, tendon reflexes, skin punctures
Biochemiacl analysis of blood and urine	Total cholesterol, LDL-cholesterol, HDL-cholesterol, triglycerides, fasting plasma glucose, alanine aminotransferase, complete blood count, urine routine test, urinary PAH metabolites, 23 urinary metals

Within 15 days after the study visiting, the participants received feedback on the results of examination (e.g. blood pressure, heart rate, hepatic function, renal function, lung function, lipids and fasting glucose) according to clinic guidelines. Meanwhile, advice or evaluation of the results were recommended by a general practitioner.

Participants are followed for physiological or pathological changes or incidence of cardiopulmonary diseases ever 3 years.

### Data collection

#### Questionnaires

Questionnaires were conducted by the trained interviewers. The general questionnaire included demographics and socioeconomics, lifestyle, family disease history (e.g. family history of hypertension, hyperlipidemia, coronary heart disease, emphysema, chronic bronchitis, asthma, and COPD), past medical history, living condition, and environmental exposure (e.g. exposure to occupational hazards such as industrial dust, noise, tobacco smoking, cooking or heating in the winter).

A revised semi-quantitative food frequency questionnaire (FFQ), based on a originally validated FFQ in the Shanghai population [29, 30], was used to assess the dietary intake. In the FFQ, we classified the food into seven food groups, rice or noodles, coarse grains (e.g. maize, barley and sweet potatoes), vegetables and fruits, dairy and eggs, meat (red meat and poultry), fishery product (fish, shrimp and crab), and pickles. For each food group, participants were asked the frequently (daily, weekly, monthly, or never) and amount which they consumed the food in each group.

The data on daily physical activity over the past six months was obtained based on the Short Questionnaire to Assess Health-enhancing physical activity (SQUASH) [31]. The sleep questionnaire was designed according to Pittsburgh Sleep Quality Index (PSQI) [32]. In the questionnaire, each participant was asked when to go to sleep and wake up, sleep latency, self-reported sleep quality (good/general/poor) and if use any sleep aid medication. The information for afternoon nap was also collected.

#### Physical examination

All participants underwent a physical examination which included anthropometry, heart rate and blood pressure measurements, and examination of tonsil, lung, heart, liver, spleen and kidney. Standing height, body weight, waist circumference, and hip circumference were measured when the participants standing with light indoor clothing and without shoes. Height and body weight were measured with the weighing scale with a vertically fixed ruler (RGZ-120, Jiangsu Suhong Medical Device Co., Ltd, Changzhou, China). We measured waist circumference with a horizontally placed tape measure mid-way between the lower costal margin and the iliac crest with a precision of one centimetre, and hip circumference at the maximum circumference of the buttocks.

Heart rate and brachial blood pressure was measured in a seated position on the right arm using a validated automatic oscillometric device (OMRON, HEM-8102A, OMRON (Dalian) Co., Ltd, Dalian, China). Participants were measured twice with five minutes rest interval. The time of lower values was accepted. The examination of tonsil, auscultation of heart and lung, and palpation of liver, spleen and kidney were performed by specialist physicians.

#### Urine sampling

Not less than 20 ml early morning urine for each participant was collected. One milliliter urine sample was analyzed for urine routine. The results included occult blood, bilirubin, ketone body, protein, nitrite, glucose, urine pH and urine-specific gravity. Other urine sample was aliquoted and stored at -20°C. We determined the concentrations of polycyclic aromatic hydrocarbons (PAHs) metabolites (using gas chromatography–mass spectrometry), 8-hydroxy-2′-deoxyguanosine (8-OHdG) (using high performance liquid chromatography with electrochemical detection), metal elements (using inductively Coupled Plasma Mass Spectrometry) using urine samples.

#### Fasting blood sampling

A total of 15 ml fasting blood sample for each participant [2 × 5 ml ethylenediamine tetraacetic acid (EDTA) anticoagulation tubes and 1 × 5 ml coagulation tube for serum] was collected. The blood lipids included total cholesterol (TC), triglycerides (TG), high-density lipoprotein cholesterol (HDL-C) and low-density lipoprotein cholesterol (LDL-C), fasting glucose, and alanine aminotransferase (ALT) were immediately determined in the clinical laboratory. The results of blood cell count, mean corpuscular volume, haemoglobin, haematocrit, mean corpuscular haemoglobin, mean corpuscular haemoglobin concentration, erythrocyte haemoglobin distribution width, platelet count and mean platelet volume, platelet volume distribution width were determined.

The remaining blood was separated into plasma (4 tubes, 500 μl per tube), serum (3 tubes, 500 μl per tube), and whole blood cells (4 tubes, 500 μl per tube), and the aliquots were stored at -80°C for future analyses. Genomic DNA for each participant was isolated and stored at -80°C for future researches.

#### Exhaled carbon monoxide

Exhaled CO was measured for each participant with a MicroCO Meter (Carefusion, Kent, UK). As exhaled CO was observed to strongly correlate with the number of cigarettes smoked in the last 24 hours [33], we asked all participants to refrain from smoking for ≥ 1 day. After a 5-minute rest, the participants were instructed to inspire fully, hold their breath for 20 seconds, then seal their lips around the mouthpiece and exhale slowly and fully. Expired alveolar gas was entrapped between mouthpiece valve and sensor for detection. Calibrations were performed with standard gas (20 ppm CO in air) on a weekly basis.

#### Exhaled nitric oxide

We measured exhaled NO following the recommendations of the American Thoracic Society/European Respiratory Society (2005) [34] with a Nano Coulomb Nitric Oxide Analyzer (SV-02E, Sunvou Medical Electronics Co., Ltd. , Wuxi, China). We asked all participants to fast overnight, refrain from smoking for ≥ 1 day. The tests were conducted at least 2 hours after food eating and 30 minutes after strenuous exercise. After being comfortably seated, the participants were instructed to inhale to total lung capacity through a NO-scrubber to eliminate ambient NO in the inhaled air, and then exhale. Exhaled NO measurements were performed at an expiratory flow rate of 50 ± 5 milliliters per second. The mean exhaled nitric oxide concentration over a 3-second plateau was defined as Exhaled NO value. Calibrations were performed with standard bottle gas (15, 75 and 150 ppb) on a weekly basis.

#### Lung function

Forced expiratory volume in one second (FEV1), forced vital capacity (FVC) and the ratio of FEV1 to FVC were determined by spirometry using a digital spirometer interfaced to a computer (Chestgraph HI-101, CHEST MI, Inc., Tokyo, Japan). All spirometric examinations were performed when the participants were in sitting position with a nose clip. Each participant was required to perform three satisfactory curves according to the recommendations of the American Thoracic Society [35], and the highest values were used in analyses. The lung function results are expressed as expiratory volume (ml) and percentage of the predicted values of individuals with similar characteristics (sex, age and height). Calibrations were performed each morning according to the manufacturer’s instruction.

#### Heart rate variability

HRV measurement methods have been described previously [36, 37]. In brief, after a 5-minute rest in seated position, each participant was fitted with a 3-channel digital electrocardiographic (ECG) monitor (Lifecard CF; Del Mar Reynolds Medical, Inc., Whitney, Irvine, USA) which ran at a sampling rate of 1024 samples/second for 10 minutes. ECG data were recorded automatically into a removable flash card. Only heart rates between 40 and 100 beats per minute were submitted to analyses. We selected 5 consecutive minutes and 24 hours (for participants in subgroup) of ECG recordings without ectopic complexes, atrial or ventricular fibrillations for HRV analysis. The HRV spectrum was computed with a fast Fourier transform method. The HRV was analyzed in both time and frequency domains. The measured time-domain parameters included standard deviation of all normal to normal intervals (SDNN) and root mean square successive difference (rMSSD). The frequency-domain variables included low frequency (LF), high frequency (HF), their ratio (LF/HF) and total power (TP).

#### Environmental exposure assessment

The concentrations of PM_10_ concentrations from 2002 to 2013 were collected from the monitoring station of Chinese Environmental Monitoring Center (CNEMC) which located within 5 km distance from the community. The data on PM_2.5_ was not available before 2013, because PM_2.5_ was routinely monitored since 2013. CNEMC is a monitoring center of the Ministry of Environmental Protection, China. It regularly published data of PM_10_ and PM_2.5_ through its monitoring network throughout China.

Outdoor particulate air pollution data on PM_10_ and PM_2.5_ for four reasons in one year (15 days in each reason) were determined by our research group through fixed-site monitoring in each community. The fixed-site monitoring equipments (TH-150C, Wuhan Tianhong Instruments Co., Ltd, Wuhan, China) were placed on the top of building (15 to 30 meters from the ground, without high-rise building surrounded) in the center of communities. Personal 24-hour PM exposures for 240 participants were monitored using personal PM_10_ and PM_2.5_ sampler (Model 200 Personal Environmental Monitor, MSP Corporation, Minnesota, U.S.A.) and pump of Gilian 5000 (Sensidyne Company, Florida, USA). The pumps for personal sampling were placed in a small backpack and PM samplers were placed at the height of the respiratory zone for each participant.

### Ascertainment of endpoints during follow-up

Self-reported chronic diseases, such as COPD, asthma, coronary heart disease (CHD ) and diabetes, are verified through medical record reviews. Participants are followed for the lung function declined, changes of HRV, hypertension, dyslipidemia, cardiovascular diseases, COPD, and asthma in medical physical examination every 3 years. Personal information, living habit, environmental exposure will be repeatly questioned during follow-up. The concentration of PM_10_, PM_2.5_ will be determined during physical examination. Earlier physiological or pathological changes were evaluated by physical examination and clinical laboratory test.

### Baseline characteristics of the enrolled population

A total of 4812 participants were included in the cohort. As shown in Table 2, the response rate for the baseline survey was 85.6% (82.6% in Wuhan city and 91.4% in Zhuhai city). The baseline characteristics of all participants were shown in Table 3. Females made up 67.5% of the participants (64.6% in Wuhan city and 72.7% in Zhuhai city). The mean age for all participant was 53.1 years (53.0 years in Wuhan city, 53.3 years in Zhuhai city). Current smoking rate was 16.5% (19.2% in Wuhan city and 11.3% in Zhuhai city) and current alcohol using rate was 13.8% (17.6% in Wuhan city and 7.3% in Zhuhai city). Among the participants, 25.3% of them did not receive secondary education and 12.5% participants finished college or higher education.

**Table 2:** **Response rates of the participants in the WHZH cohort**

	Cohort	Wuhan	Zhuhai
Invited	5622	3698	1924
Completed	4812	3053	1759
Response rate	85.6%	82.6%	91.4%

**Table 3:** **The baseline characteristics of all participants in the WHZH cohort**

Variables	Total (n = 4812)	Wuhan (n = 3053)	Zhuhai (n = 1759)
**Gender, (female, n (%))**	3252 (67.6)	1973 (64.6)	1279 (72.7)
**Age (years)**	53.1 (13.0)	53.0 (13.3)	53.3 (12.4)
**Age (years), n (%)**			
18-44	1256 (26.1)	796 (26.1)	460 (26.2)
45-59	2068 (43.0)	1315 (43.1)	753 (42.8)
60-80	1488 (30.9)	942 (30.9)	546 (31.0)
**Education, n (%)**			
Primary school or illiteracy	1219 (25.3)	644 (21.1)	575 (32.7)
Middle school	1655 (34.4)	1067 (34.9)	588 (33.4)
High school	1338 (27.8)	965 (31.6)	373 (21.2)
University or college or higher	600 (12.5)	377 (12.3)	223 (12.7)
**Income (Yuan/year)** ^**#**^ **, n (%)**			
≤39999	2642 (54.9)	1856 (60.8)	786 (44.7)
40000-119999	1886 (39.2)	1104 (36.2)	782 (44.5)
≥120000	201 (4.2)	57 (1.9)	144 (8.2)
Missing, n (%)	83 (1.7)	36 (1.2)	47 (2.7)
**Current smoker, n (%)**	795 (16.5)	597 (19.2)	198 (11.3)
**Current drinker, n (%)**	666 (13.8)	537 (17.6)	129 (7.3)
**Physical activity, (yes, n (%))**	2346 (48.8)	1353 (44.3)	993 (56.4)
**BMI (kg/m** ^**2**^ **)**	24.0 (3.5)	24.1 (3.4)	23.7 (3.5)
**Waist circumference (cm)**	818.7 (97.6)	820.9 (97.7)	814.8 (97.3)
**Blood pressure (mmHg)**			
Systolic blood pressure	131.5 (20.3)	131.0 (20.5)	132.4 (20.0)
Diastolic blood pressure	77.4 (15.0)	76.6 (16.8)	78.7 (11.2)
Missing, n (%)	60 (1.2)	37 (1.2)	23 (1.3)
**Exhaled carbon monoxide (μg/L)**	7.7 (9.9)	8.5 (11.0)	6.3 (7.4)
Missing, n (%)	27 (0.6)	0 (0%)	27 (1.5)
**Exhaled nitrogen oxide (ppb)**	24.9 (17.3)	27.2 (16.3)	20.9 (18.4)
Missing, n (%)	228 (4.7)	121 (4.0)	107 (6.1)
**Lung function**			
FVC (ml)	2479.6 (698.9)	2566.5 (711.7)	2327.9 (649.0)
FEV_1_ (ml)	2159.4 (610.7)	2200.8 (615.1)	2087.2 (596.4)
FEV_1_/FVC (%)	87.5 (8.9)	86.9 (9.2)	89.9 (7.7)
Missing, n (%)	107 (2.2)	62 (2.0)	45 (2.6)
**Heart rate variable**			
TP (msec^2^)	809.4 (461.8, 1413.8)	818.0 (464.8, 1413.6)	798.8 (450.9, 1417.3)
LF (msec^2^)	218.8 (107.1, 418.4)	219.0 (107.7, 426.2)	218.8 (106.3, 406.2)
HF (msec^2^)	119.7 (56.4, 250.8)	125.8 (59.9, 259.9)	110.8 (51.1, 234.6)
Missing, n (%)	315 (6.5)	203(6.6)	112(6.4)

Variables are presented as mean ± SD, percentage or median (25th, 75th quartile). BMI, body mass index; FVC, forced vital capacity; FEV_1_, forced expiratory volume in 1 second; TP, total power; LF, low frequency; HF, high frequency. ^#^Chinese RMB.

The mean BMI was 24.0 kg/m^2^ (24.1 kg/m^2^ for males, 23.9 kg/m^2^ for females) and the mean waist circumference was 818.7 cm (852.8 cm for males, 802.3 cm for females). The mean systolic and diastolic blood pressures were 131.5 mmHg and 77.4 mmHg, respectively. Among participants, the mean exhaled carbon monoxide and exhaled nitrogen oxide were 7.7 μg/l (8.5 μg/l in Wuhan city and 6.3 μg/l in Zhuhai city) and 24.9 ppb (27.2 ppb in Wuhan city and 20.9 ppb in Zhuhai city), respectively. The mean values for FVC, FEV1 were 2479.6 ml and 2159.4 ml. The FEV1/FVC was 87.5%. The median TP, LF, and HF were 809.4 mes^2^, 218.8 mes^2^, and 119.7 mes^2^, respectively. The mean values of clinic biochemical parameters including hepatic function parameters, lipids, fasting glucose and haematological trait were displayed in Table 4. The mean values of TC, TG, HDL-C and LDL-C were 5.1, 1.5, 1.6 and 3.1 mmol/l, respectively. The prevalence of self-reported diseases was shown in Table 5. The prevalence of self-reported hypertension and hyperlipidemia was 20.8% and 6.8%, respectively. The prevalence of self-reported chronic bronchitis and asthma was 4.9% and 1.1%, respectively.

**Table 4:** **The baseline levels of biochemical traits of the participants in the WHZH cohort (mean ± SD)**

Variables	Male	Female	Total
**ALT (U/l)**	27.1 (19.1)	20.7 (14.8)	22.8 (16.6)
**TC (mmol/l)**	5.0 (1.4)	5.2 (1.2)	5.1 (1.3)
**TG (mmol/l)**	1.6 (1.3)	1.5 (1.2)	1.5 (1.2)
**HDL-C (mmol/l)**	1.5 (0.9)	1.6 (0.4)	1.6 (0.6)
**LDL-C (mmol/l)**	3.1 (1.0)	3.1 (1.0)	3.1 (1.1)
**Fasting glucose (mmol/l)**	5.0 (1.7)	4.8 (1.5)	4.9 (1.6)
**RBC (t/l)**	4.7 (0.6)	4.4 (0.7)	4.5 (0.7)
**WBC (g/l)**	6.2 (2.5)	5.8 (2.1)	5.9 (2.2)
**Platelet count (g/l)**	212.4 (64.9)	223.7 (62.6)	220.1 (63.6)
**Hemoglobin (g/l)**	148.5 (17.9)	133.0 (16.8)	138.0 (18.6)

ALT, alanine aminotransferase; TC, total cholesterol; TG, triglycerides; HDL-C, high density lipoprotein cholesterol; LDL-C, low density lipoprotein cholesterol; RBC, red blood cell count; WBC, white blood cell count.

**Table 5:** **The prevalence of self-reported diseases in the WHZH cohort**

Variables	Total (n = 4812)	Wuhan (n = 3053)	Zhuhai (n = 1759)
**Hypertension, n (%)**	1000 (20.8)	693 (22.7)	307 (17.5)
**Hyperlipidemia, n (%)**	325 (6.8)	228 (7.5)	97 (5.5)
**Diabetes mellitus, n (%)**	288 (6.0)	200 (6.6)	88 (5.0)
**Angina, n (%)**	57 (1.2)	40 (1.3)	17 (1.0)
**Myocardial infarction, n (%)**	31 (0.6)	22 (0.7)	9 (0.5)
**Stroke, n (%)**	55 (1.1)	44 (1.4)	11 (0.6)
**Chronic bronchitis, n (%)**	238 (4.9)	199 (6.5)	39 (2.2)
**Asthma, n (%)**	54 (1.1)	39 (1.3)	15 (0.8)
**Pulmonary tuberculosis, n (%)**	70 (1.5)	60 (2.0)	10 (0.6)
**Chronic hepatitis, n (%)**	69 (1.4)	55 (1.8)	14 (0.8)
**Nephritis, n (%)**	58 (1.2)	52 (1.7)	6 (0.3)

## Discussion

### PM exposure assessment

Environmental PM levels and personal PM exposure were assessed in this study. Data of environmental PM levels come from Governmental Environmental Monitoring Center nearby the communities, direct monitoring of outdoor PM_2.5_ and PM_10_ inside the communities. Personal PM exposure were collected on 240 randomly selected persons. The PAHs and 23 metals in PM_2.5_ were also directly determined on the filters of environmental PM samplers and all personal samples. All these information will be used to establish an environmental PM_10_ or PM_2.5_ and calendar year exposure matrix and to estimate PM and its composition exposure for each participant. The possible influence of indoor cooking, smoking or traffic factors will be evaluated.

### Pathophysiological changes

In this study, declined lung function and changes of HRV indices were used as the earlier endpoints of cardiopulmonary damage. Lung function has been known as predictor of cardiorespiratory health and longevity and physical activity [38–40] although the causal pathway of lung function alteration is poorly understood [41]. Previous studies reported that decreases in the ratio of FEV1 to FVC and FEV1 were associated with increased exposure to particulate matter in nonsmoking young men and dust exposed workers [42–44]. Other studies also reported decrease in lung function were associated with high particulate air pollution among children [45, 46]. HRV is used to assess the autonomic modulation of cardiac rhythm and quantitatively estimate cardiac autonomic activity [47]. And impaired cardiac autonomic functions were associated with increased risk for cardiovascular diseases (CVDs) [48, 49]. Therefore, altered HRV were thought to be a earlier sign for cardiovascular disease events [50].

### Advantage and limitation

There are several advantages of this study. Firstly, two cities with similar life style and significant different ambient PM exposure will provide a great opportunity to observe adverse health effects from ambient PM on general population. Secondly, not only ambient PM exposure, we also estimated personal PM exposure in this study. It could provided accurate exposure-effects relationship between PM exposure and health damage over follow-ups. Finally, the members of community office will help us to minimize loss to follow-up.

A weakness of this cohort study is that the percent of middle-age and older adults in this study is higher than general population composition. And female participants percent is higher than 60%, which is little higher than mean percent of female in general population (48.7%) [51]. The possible reason for this imbalance is that young people are too busy to participate this study. However, older and female participants were thought be more vulnerable to ambient PM in previous study.

In summary, the results obtained in WHZH cohort study may increase a better understanding of the relationship between particulate air pollution and its components and possible health damages. And the potential mechanisms underlying the development of cardiopulmonary diseases has implications for the development of prevention and treatment strategies.

## Abbreviations

ALT: Alanine aminotransferase
CHD: Coronary heart disease
CNEMC: China Environmental Monitoring Center
CO: Carbon monoxide
COPD: Chronic obstructive pulmonary disease
CVDs: Cardiovascular diseases
ECG: Electrocardiograph
EDTA: Ethylenediamine tetraacetic acid
FEV1: Forced expiratory volume in one second
FFQ: Food frequency questionnaire
FVC: Forced vital capacity
HDL-C: High density lipoprotein cholesterol
HRV: Heart rate variability
HF: High frequency
LDL-C: Low density lipoprotein cholesterol
LF: Low frequency
NO: Nitric oxide
PAHs: Polycyclic aromatic hydrocarbons
PM: Particulate matter
PM_10_: Particles with aerodynamic diameters less than 10 μm
PM_2.5_: Particles with aerodynamic diameters less than 2.5 μm
PSQI: Pittsburgh Sleep Quality Index
rMSSD: Root mean square successive difference
SDNN: Standard deviation of all normal to normal intervals
SQUASH: Short Questionnaire to Assess Health-enhancing physical activity
TC: Total cholesterol
TF: Total frequency
TG: Triglycerides
WHO: World Health Orgnization.
